# Neuronal Avalanches to Study the Coordination of Large-Scale Brain Activity: Application to Rett Syndrome

**DOI:** 10.3389/fpsyg.2020.550749

**Published:** 2020-10-27

**Authors:** Rosaria Rucco, Pia Bernardo, Anna Lardone, Fabio Baselice, Matteo Pesoli, Arianna Polverino, Carmela Bravaccio, Carmine Granata, Laura Mandolesi, Giuseppe Sorrentino, Pierpaolo Sorrentino

**Affiliations:** ^1^Department of Motor Sciences and Wellness, University of Naples “Parthenope,” Naples, Italy; ^2^Institute of Applied Sciences and Intelligent Systems, National Research Council (CNR), Pozzuoli, Italy; ^3^Department of Medical and Translational Science, Child Neuropsychiatry Unit, University of Naples “Federico II,” Naples, Italy; ^4^Department of Neuroscience, Pediatric Psychiatry and Neurology, Santobono-Pausilipon Children’s Hospital, Naples, Italy; ^5^Department of Engineering, University of Naples “Parthenope,” Naples, Italy; ^6^Hermitage Capodimonte Hospital, Naples, Italy; ^7^Department of Humanistic Studies, University of Naples “Federico II,” Naples, Italy; ^8^Institut de Neurosciences des Systèmes, Aix-Marseille Université, Marseille, France

**Keywords:** self-organized criticality, critical state, neuronal avalanches, branching process, magnetoencephalography, Rett syndrome

## Abstract

Many complex systems, such as the brain, display large-scale coordinated interactions that create ordered patterns. Classically, such patterns have been studied using the framework of criticality, i.e., at a transition point between two qualitatively distinct patterns. This kind of system is generally characterized by a scale-invariant organization, in space and time, optimally described by a power-law distribution whose slope is quantified by an exponent α. The dynamics of these systems is characterized by alternating periods of activations, called avalanches, with quiescent periods. To maximize its efficiency, the system must find a trade-off between its stability and ease of propagation of activation, which is achieved by a branching process. It is quantified by a branching parameter σ defined as the average ratio between the number of activations in consecutive time bins. The brain is itself a complex system and its activity can be described as a series of neuronal avalanches. It is known that critical aspects of brain dynamics are modeled with a branching parameter σ = , and the neuronal avalanches distribution fits well with a power law distribution exponent α = -3/2. The aim of our work was to study a self-organized criticality system in which there was a change in neuronal circuits due to genetic causes. To this end, we have compared the characteristics of neuronal avalanches in a group of 10 patients affected by Rett syndrome, during an open-eye resting-state condition estimated using magnetoencephalography, with respect to 10 healthy subjects. The analysis was performed both in broadband and in the five canonical frequency bands. We found, for both groups, a branching parameter close to 1. In this critical condition, Rett patients show a lower distribution parameter α in the delta and broadband. These results suggest that the large-scale coordination of activity occurs to a lesser extent in RTT patients.

## Introduction

Rett syndrome (RTT) is a severe neurodevelopmental disorder, characterized by clinically normal development for the first 12–18 months of life, when an overall arrest and regression of the psychomotor development begins. The patients lose the acquired verbal skills, the normal motor hand function is replaced with stereotyped movements, and often ataxia is present, which seriously compromises motor skills. Intellectual disability, epilepsy, autonomic dysfunction, breathing abnormalities, anxiety, and orthopedic problems are the other most important clinical findings in RTT (Neul et al., 2010). In 1999, Amir et al. (1999) identified in a mutation in the X-linked MECP2 gene, encoding the methyl CpG-binding protein 2 (MeCP2), the most common cause of RTT.

The dysfunction of *MeCP2* provokes multiple effects, such as impaired neuronal maturation, altered GABAergic signaling, and, more importantly, a local imbalance between neuronal excitation and inhibition at the circuit level (Pohodich and Zoghbi, 2015). Importantly, all these molecular and circuital evidence are scarcely linked to the clinically evident deficits in higher cognitive functions. In fact, effective coordination of large-scale activity is considered necessary for the emergence of high-level cognitive abilities. All these features make RTT a potential model for studying how an alteration of the normal development process interferes with the activity of brain networks.

Consequently, we borrowed from physics some tools that allow the description of large-scale activity. It has been shown that the healthy brain has a higher number of configurations compared to the unhealthy brain (Sorrentino et al., 2018). Importantly, it has been argued that flexibility is possible given that the brain sets itself to operate near a critical state (Beggs and Plenz, 2003). Bak et al. (1987) first described the idea of “self-organized criticality” (SOC) defined as a system autonomously evolving to a critical state regardless of the initial condition. As he defines, “the critical state is an attractor for the dynamics of a system.” In the critical state, the activity can spread across the system in a controlled way, avoiding that activations either spread uncontrollably or quickly die out (Zapperi et al., 1995; Harris, 2002). Such state is classically quantified by a branching parameter σ defined as the average ratio between the number of activations in consecutive time bins (i.e., the sample units) (De Carvalho and Prado, 2000). More specifically, when σ < 1, the system is in a subcritical condition in which the activations quickly fade out. On the other hand, when σ > 1, the opposite happens, and the system is in a supercritical state whereby the activations spread across the entire system, leading to unstable runaway activations. Finally, when σ = 1, the system is in a critical state with balanced, fine-tuned propagation of the activity (Bak et al., 1987; Harris, 2002).

SOC systems are generally characterized by a scale-invariant organization in space and time. In other words, the fluctuations that occur are similar in all scales of time and space, and they can be optimally described by fractal metrics, typically a power law distribution whose slope quantifies the amplitude distribution of such fluctuations (Paczuski et al., 1996; Newman, 2007; Clauset et al., 2009).

Importantly, power law distributions do not prove criticality (Clauset et al., 2009). However, they do provide a statistical measure of the occurrence of large-scale coordinated activity, perhaps providing a useful tool to probe hypothesis about the nature of high-level cognitive deficits, such as those observed in Rett syndrome.

Generally speaking, SOC can be found in very different types of phenomena, like in many natural systems such as earthquakes (Gutenberg and Richter, 1956) or forest fires (Malamud et al., 1998), in which when a unit exceeds a threshold, other units follow it, thus giving rise to a cascade (or avalanche) that spreads across the entire system. The presence of power law-distributed bursts of activations, called avalanches, alternating with quiescent periods, characterizes the dynamics of SOC systems (Lombardi et al., 2016) likely maximizing the efficiency of communication among its elements. However, it is to be considered that the analysis of avalanches, with a power law distribution, varies in heterogeneous/disordered systems and that to get information on the non-equilibrium dynamics, it would be useful to also analyze the crackling noise (Salje and Dahmen, 2014).

In this article, we hypothesize that the genetic defect causing RTT impairs the whole-brain dynamic and relates to clinical symptoms. To test our hypothesis, we compared the distributions of the size of the neuronal avalanches, in order to capture the critical features of brain activity, in 10 patients with RTT and 10n matched healthy subjects (HS). We expect that large-scale activation would be occurring less in patients showing marked deficits in higher cognitive functions. We based our analyses on source-reconstructed magnetoencephalographic (MEG) data acquired during resting state.

## Materials and Methods

### Participants and Clinical Assessment

Participants were recruited from the Department of Translational Medical Sciences, Child Neuropsychiatry in “Federico II” University of Naples, Italy. We studied 10 female patients with clinical diagnosis of RTT (age 24.30 ± 8 years) based on the Neul revised criteria (Neul et al., 2010) and confirmed by mutation in the *MECP2* gene, and 10 healthy female individuals (HS) (age 26.10 ± 6.84 years). This study complied with the Declaration of Helsinki and was approved by the local ethics committee. Written informed consent has been granted by all participants (or their legal guardians).

### Acquisition

The data were acquired using a MEG system equipped by 163 magnetometers SQUID (Superconducting Quantum Interference Device) (Sorrentino et al., 2018), placed in a magnetically shielded room (AtB Biomag, Ulm, Germany), in order to reduce the external noise. Of all squids, 154 are positioned to be as close as possible to the head of the subject, while the remaining ones are more distant so as to measure environmental noise (reference magnetometers). All the subjects of each group underwent a 7-min resting-state MEG acquisition with open eyes, divided in two segments. To evaluate the right position of the head under the helmet, we used Fastrak (Polhemus^®^) to acquire the position of four coils (attached to the head) and of four anatomical landmarks (nasion, right and left pre-auricular points, and vertex of the head). Before each acquisition segment, the head position in the helmet was obtained. During the acquisition, two electrodes for the electrocardiogram and two for the electrooculogram (Gross et al., 2013) were also acquired.

### Preprocessing

As previously described (Jacini et al., 2018; Rucco et al., 2019), the MEG signals, after an anti-aliasing filter, were acquired with a sampling frequency of 1,024 Hz. A fourth-order Butterworth IIR band-pass filter in the 0.5- to 100-Hz band was subsequently applied to the acquired signals. Environmental noise, measured by reference magnetometers, was removed by using the principal component analysis. MEG data were cleaned of physiological artifacts, such as eye blinking and heart activity, by means of independent component analysis (Sorriso et al., 2019). Visual inspection was used for identification of noisy channels. For all the preprocessing steps, we used the FieldTrip toolbox (Oostenveld et al., 2011).

### Source Reconstruction

Time series of neuronal activity were reconstructed in 116 regions of interests (ROIs) based on the automated anatomical labeling (AAL) atlas (Tzourio-Mazoyer et al., 2002) using a linearly constrained minimum variance beam-former algorithm (Van Veen et al., 1997; Nolte, 2003) based on MRI template and then filtered both in broadband (0.5–48 Hz) and in the five classical frequency bands [delta (0.5–4.0 Hz), theta (4.0–8.0 Hz), alpha (8.0–13.0 Hz), beta (13.0–30.0 Hz), and gamma (30.0–48.0 Hz)]. From the 116 ROIs of the AAL Atlas, we have excluded 26 ROIs corresponding to the cerebellum because of their low reliability in MEG (Lardone et al., 2018). Hence, we considered a total of 90 ROIs. We have resampled the source-space time series at 512 Hz.

### Signal Discretization

For each ROI, the time series were discretized with five different time bin durations Δ*t*, each one multiple of Δ*t*_*min*_ (19 ms), corresponding to the sampling period.

An event was identified by a positive or a negative excursion of an area, in a bin, beyond a threshold, defined as ±3 SD of the signal amplitude, which was a tradeoff between a lower threshold (leading to the detection of more spurious noise events in addition to real ones) and a higher threshold (missing real events). Indeed, MEG systems rely on squid sensors, which measure the magnetic field generated by brain activity. However, such sensors cannot identify the field direction. For this reason, both positive and negative excursions were picked up without distinction. As a result, the event was identified when the absolute value of this excursion exceeded the chosen threshold.

### Branching Parameter

As previously described (Beggs and Plenz, 2003; Shriki et al., 2013), an avalanche is defined as a sequence of contiguous time bins starting when at least one ROI is active and ending when all regions are inactive. The number of events in all ROIs in an avalanche corresponds to its size.

For each subject and for each time bin size, the branching parameter σ was estimated by calculating, for each avalanche, the averaged (over all the time bins) ratio of the number of events between the subsequent time bin (descendants) and that in the current time bin (ancestors), and then averaging it over all the cascades (Bak et al., 1987). More specifically:

(1) $$\sigma =\frac{1}{N_{a\,v\,a\,l}}\,\sum_{i=1}^{N_{a\,v\,a\,l}}\sigma_{i}$$

(2) $$\sigma_{i}=\frac{1}{N_{b\,i\,n}-1}\,\sum_{j=1}^{N_{b\,i\,n}-1}\frac{n_{e\,v\,e\,n\,t\,s}\,\left(j+1\right)}{n_{e\,v\,e\,n\,t\,s}\,\left(j\right)}$$

where σ_*i*_ is the branching parameter of the *i*-th avalanche in the dataset, *N*_*bin*_ is the total amount of bins in the *i*-th avalanche, *n*_*events*_(*j*) is the total number of events active in the *j*-th bin, and *N*_*aval*_ is the total number of avalanche in the dataset.

### Neuronal Avalanches and Power Law Parameter

**FIGURE 1:**
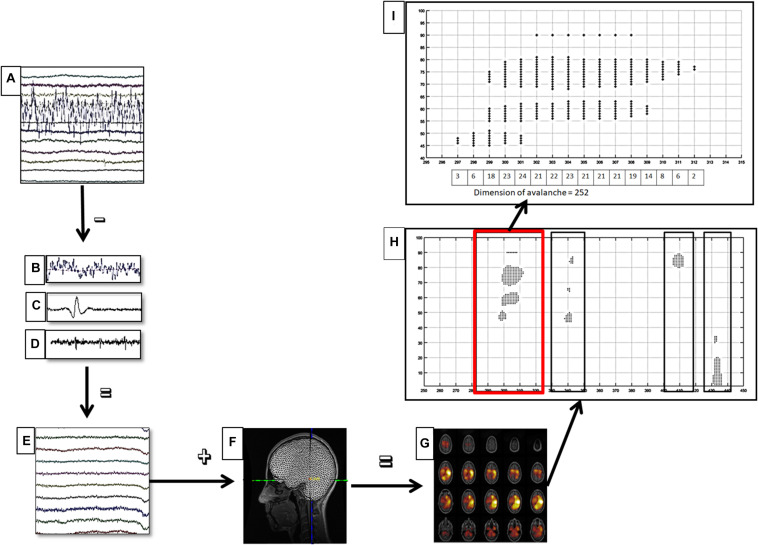
Data analysis pipeline. **(A)** Raw magnetoencephalography (MEG) signals recorded by 154 sensors (a subset displayed here). **(B–D)** Respectively noisy channel, cardiac artifact, blinking artifact, removed during preprocessing phase. **(E)** MEG signals after artifact removal and noise cleaning. **(F)** Coregistration between MEG signals and MRI template. **(G)** Source reconstruction (beamforming). **(H)** A sequence of four neuronal avalanches. **(I)** How to calculate the dimension of a neuronal avalanche, in particular, the first (in red square) of the **(H)** image.

The statistical distribution of the avalanche size *x* was assumed to be described by a power law function:

(3) $$P_{\alpha }=\left\{ \begin{array}{c} C_{\alpha }\,x^{\alpha }\;x_{m\,i\,n}\leq x\leq x_{m\,a\,x}\\ 0\;o\,t\,h\,e\,r\,w\,i\,s\,e \end{array}\right.$$

where *C*_α_ is a normalization factor. The parameters *x*_*min*_ was set to 1 (the minimal avalanche size), and *x*_*max*_ was set to 1.5 times the total number of ROIs included in the analysis. We used truncated power law to take finite size effect into account. The distribution parameter α was estimated from the data by implementing a maximum likelihood algorithm (Kay, 1993). When the branching parameter σ is equal to 1, the system is in a critical state. In other words, there is a long distance propagation of neuronal activity without runaway excitation. For this reason, we calculated at which time bin, for each frequency band, the branching parameter (as average distance) was the closest to 1 for both groups. Such choice was made in order to have the two groups in the same condition, in order to allow a meaningful comparison of the α parameter. Furthermore, to evaluate the robustness of our data, we split our dataset both in two and in three segments, and for each of them, we calculated the sigma parameter and his variance. Figure 1 shows the data analysis pipeline.

### Goodness of Fit

We performed the goodness of fit (GOF) via the evaluation of Hellinger distance (HD) (Oosterhoff and van Zwet, 2012) between the distribution of real data and the distribution of fitted data, in order to quantify the similarity between two probability distributions, as follows.

(4) $$H\,D\,\left(P,Q\right)=\frac{1}{\sqrt{2}}\,\sqrt{\sum_{i=x_{0}}^{x_{m}}{\left(\sqrt{P_{i}}-\sqrt{Q_{i}}\right)}^{2}}$$

where *P* is the probability mass function of real data, and *Q* is the probability mass function of fitted data.

The HD is in between 0 and 1; and the lower the HD, the better the fit to real data (ideally, HD = 0). Furthermore, to assess the GOF overall RTT patients and HS, we provided the empirical cumulative distribution function (ECDF) of HD.

### Statistical Analysis

We compared the distribution parameter α in patients and controls using permutation testing (Nichols and Holmes, 2002), for each frequency band. Specifically, at each iteration, each subject is randomly assigned to one of the two groups and then the difference between the averages of the two groups was computed. The group assignment is permuted 10*4* times, obtaining the null distribution of group differences, which was used to define the statistical significance of the observed difference between patients and controls. All the analyses were performed at a significance level of 0.05, using Matlab R2017a (MathWorks^®^) environment.

## Results

As described in the Methods section, we discretized the ROI signals as a train of events, each of them representing the signal exceeding the threshold of ±3 SD.

First, we checked the critical condition in both groups, in all frequency bands, for all time bins.

We found, for both groups, a branching parameter σ≈ 1, in delta band for bin Δ*t* = 5, in theta and alpha band for bin Δ*t* = 1, in beta-, gamma-, and broadband for bin Δ*t* = 2, as reported in Table 1. Moreover, to demonstrate the robustness of the estimation of the branching parameter, we estimated the variance around sigma both when we split our dataset into two segments (Table 2) and when we divided it into three segments (Table 3).

**TABLE 1:** The mean value, for Rett syndrome (RTT) patients and health subject (HS) group, of branching parameter σ, for all frequency bands in all time bins.

	**Δ *t* = 1**		**Δ *t* = 2**		**Δ *t* = 3**		**Δ *t* = 4**		**Δ *t* = 5**	
	**RTT patients**	**HS**	**RTT patients**	**HS**	**RTT patients**	**HS**	**RTT patients**	**HS**	**RTT patients**	**HS**
Delta band	0.900	0.901	0.932	0.911	0.943	0.919	0.955	0.971	**1.031**	**1.016**
Theta band	**0.911**	**0.964**	1.137	1.179	1.139	1.226	1.189	1.286	1.270	1.329
Alpha band	**0.974**	**0.964**	1.084	1.095	1.128	1.164	1.135	1.171	1.219	1.231
Beta band	0.835	0.848	**0.981**	**0.985**	1.071	1.104	1.106	1.172	1.098	1.179
Gamma band	0.848	0.865	**1.003**	**1.041**	1.091	1.169	1.159	1.244	1.167	1.244
Broadband	0.925	0.942	**1.021**	**1.043**	1.095	1.097	1.251	1.192	1.227	1.261

*In bold are reported the time bin chosen for each frequency band for the further analysis.*

**TABLE 2:** To appreciate the robustness of the results presented, we reported the variance values around the branching parameters, for RTT patients and HS group, when dataset is split in two segments.

	**Segment 1/2**		**Segment 2/2**	
	**RTT patients**	**HS**	**RTT patients**	**HS**
Delta band (Δ*t* = 5)	1.034 (0.007)	1.021 (0.003)	1.028 (0.002)	1.011 (0.004)
Alpha band (Δ*t* = 1)	0.973 (0.001)	0.966 (0.001)	0.975 (0.001)	0.962 (0.000)
Broadband (Δt = 2)	1.018 (0.010)	1.045 (0.001)	1.024 (0.001)	1.041 (0.001)

*For brevity, we reported only delta, alpha, and broadbands.*

**TABLE 3:** To appreciate the robustness of the results presented, we reported the variance values around the branching parameters, for RTT patients and HS group, when dataset is split in three segments.

	**Segment 1/3**		**Segment 2/3**		**Segment 3/3**	
	**RTT patients**	**HS**	**RTT patients**	**HS**	**RTT patients**	**HS**
Delta band (Δ*t* = 5)	1.030 (0.010)	1.020 (0.005)	1.033 (0.004)	1.016 (0.009)	1.029 (0.005)	1.011 (0.002)
Alpha band (Δt = 1)	0.971 (0.002)	0.966 (0.001)	0.974 (0.001)	0.961 (0.000)	0.976 (0.001)	0.965 (0.001)
Broadband (Δ*t* = 2)	1.018 (0.001)	1.040 (0.002)	1.020 (0.002)	1.046 (0.001)	1.024 (0.001)	1.043 (0.002)

*For brevity, we reported only delta, alpha, and broadbands.*

**FIGURE 2:**
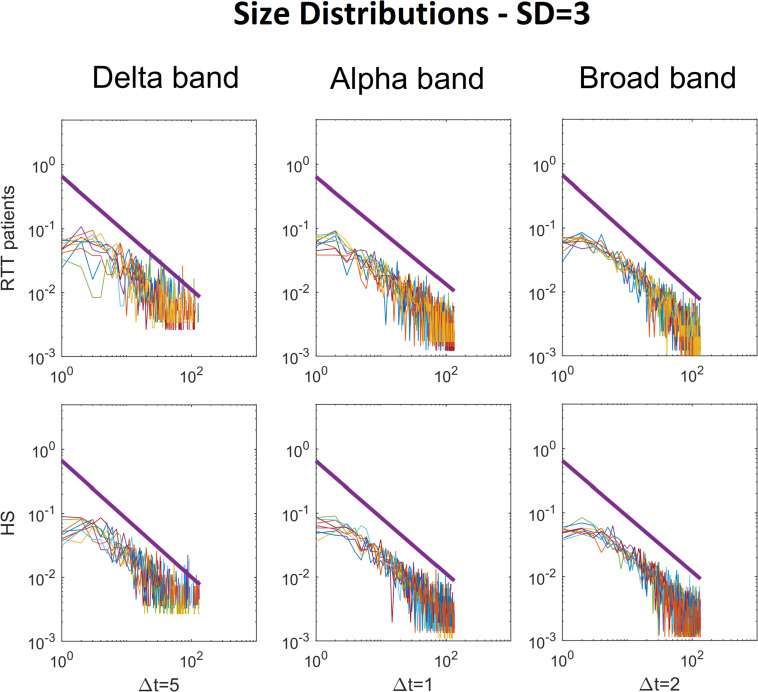
Size distributions of avalanches with the relative power law fitted, for each RTT patient (first line) and for each HS (second line). The colored lines represent the size distributions of avalanches for each subject (RTT patients in the top row, Healthy Subjects in the bottom row). The black bold lines represent the fitted power laws. For brevity, we reported only Delta (Δ*t* = 5), Alpha (Δ*t* = 1) and Broad (Δ*t* = 2) bands. Specifically, the Delta band and the Broad band reached statistical significance, whereas the Alpha band was reported for comparison, as an example, as it does not reach significance (such as Theta, Beta and Gamma). The reported results refer to the threshold SD = 3.

**FIGURE 3:**
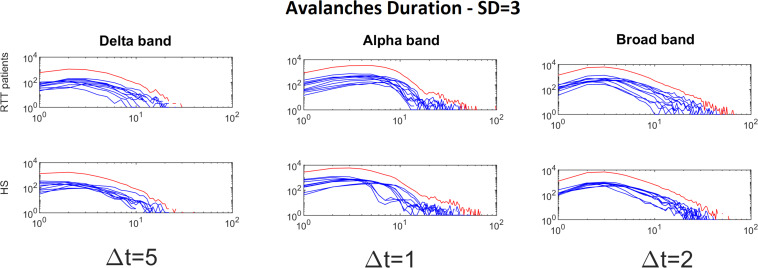
Avalanche duration distributions for each RTT patient (first line) and for each HS (second line) for Delta (Δ*t* = 5), Alpha (Δ*t* = 1) and Broad (Δ*t* = 2) bands (the threshold chosen is SD = 3). The blue lines represent the avalanche duration distributions for each subject (RTT patients in the top row, Healthy Subjects in the bottom row). The red lines represent the sum of the avalanche duration distributions across all subjects.

**FIGURE 4:**
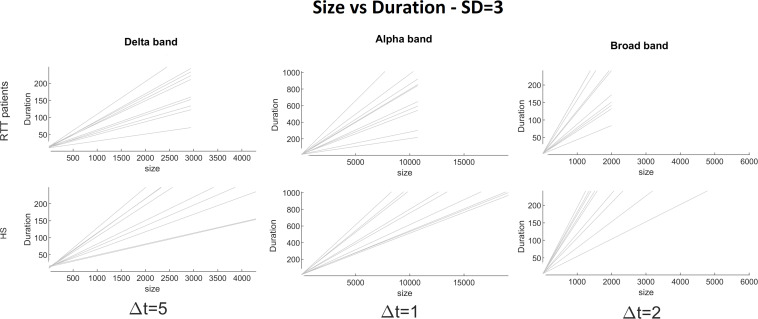
Size versus duration for each RTT patient (first line) and for each HS (second line) for delta (Δ*t* = 5), alpha (Δ*t* = 1), and broad (Δ*t* = 2) bands (the threshold chosen is SD = 3).

**FIGURE 5:**
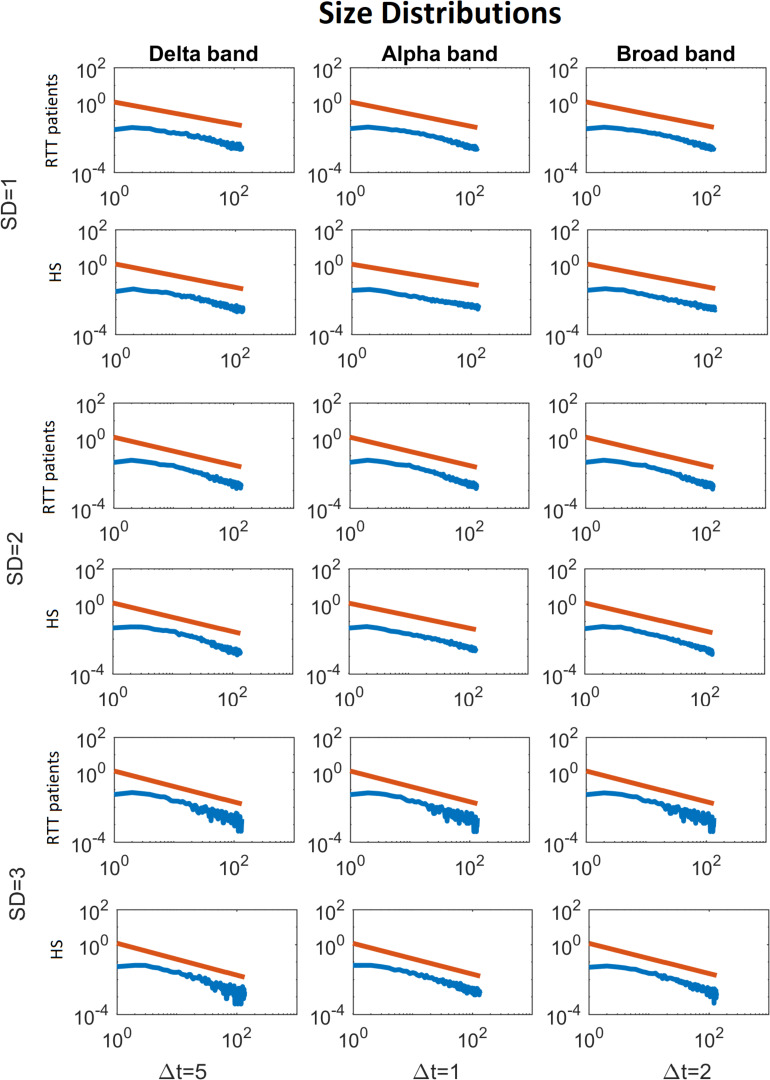
Average size distributions for both groups, for Delta (Δ*t* = 5), Alpha (Δ*t* = 1) and Broad (Δ*t* = 2) bands, as a function of the threshold (from 1 to 3 SD). The blue lines represent the average of the avalanche size distributions across either the RTT patients or the HS (as indicated in the y-label). The red lines represent the fitted power law.

**FIGURE 6:**
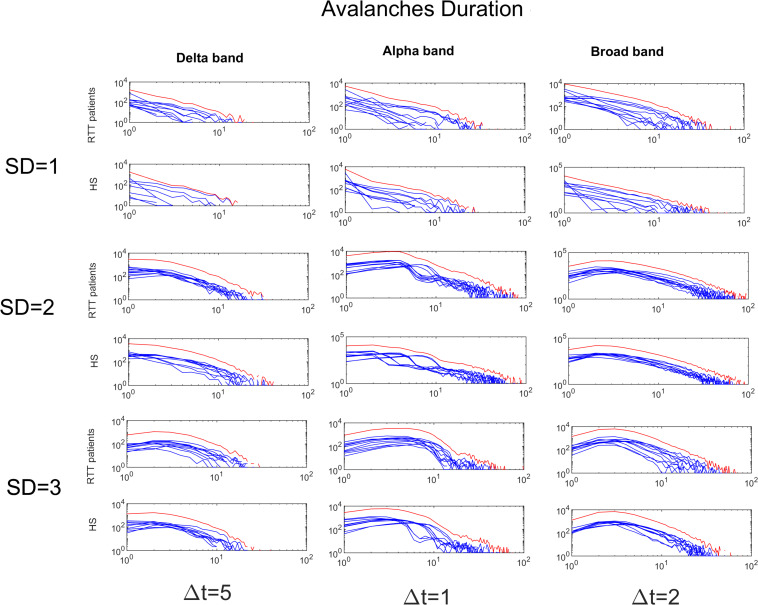
Avalanches duration distributions for both groups for Delta (Δ*t* = 5), Alpha (Δ*t* = 1) and Broad (Δ*t* = 2) bands as a function of the threshold (from 1 to 3 SD). The blue lines represent the avalanche duration distributions across either the RTT patients or the HS (as indicated in the y-label). The red lines represent the sum of the avalanche duration distributions across all subjects.

**FIGURE 7:**
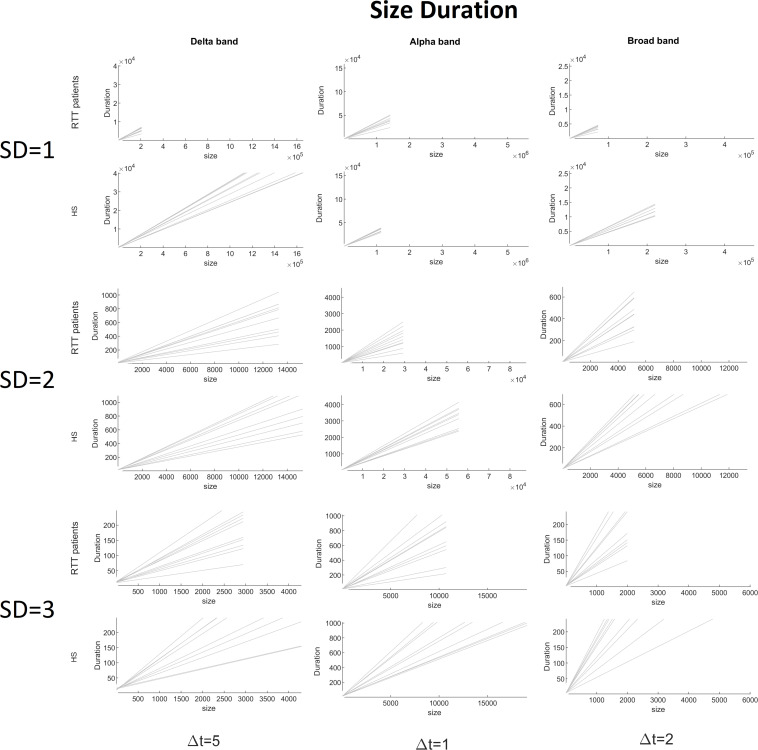
Size versus duration for both groups for delta (Δ*t* = 5), alpha (Δ*t* = 1), and broad (Δ*t* = 2) bands varying the threshold (1 to 3 SD).

To demonstrate the strength of our data, we reported, for the chosen threshold SD = 3, the size distribution fitted with a power law (Figure 2), duration distributions (Figure 3), and average size versus duration distributions (Figure 4) for each subject, in all time bins, for both groups separately (for brevity, we reported only delta, alpha, and broadbands). Again, we provided the effect of different thresholds (1 to 3 SD) on our data in size distributions (Figure 5), duration distribution (Figure 6), and average size versus duration distributions (Figure 7).

**FIGURE 8:**
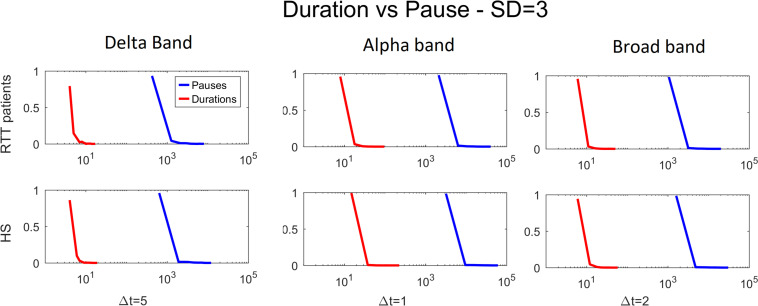
Duration versus pause for both groups for delta (Δ*t* = 5), alpha (Δ*t* = 1), and broad (Δ*t* = 2) bands varying the threshold (1 to 3 SD).

Furthermore, to test that our results might not be biased by spatial subsampling, we demonstrate the presence of scale time separation (Levina and Priesemann, 2017), as reported in Figure 8.

**FIGURE 9:**
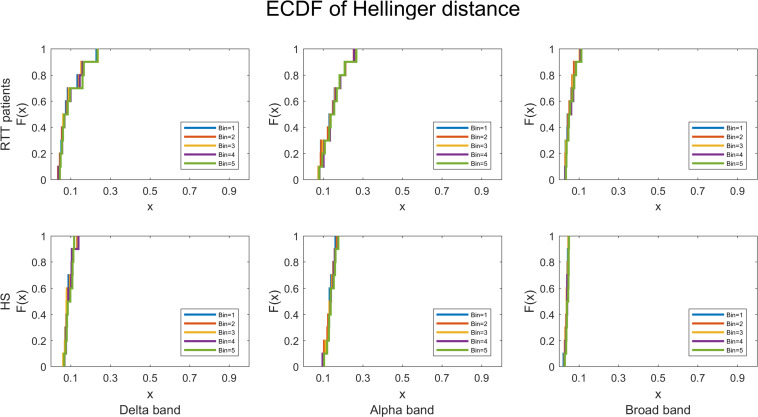
The empirical cumulative distribution functions of the Hellinger distance for RTT patients and HS separately, in all time bins, for delta, alpha, and broadbands. The chosen threshold is SD = 3.

Last, to quantify the robustness of power law fitting, we evaluated the ECDF of HD for both groups, for all time bins in delta, alpha, and broadbands, and as reported in Figure 9, the highest HD is less than 0.3.

**FIGURE 10:**
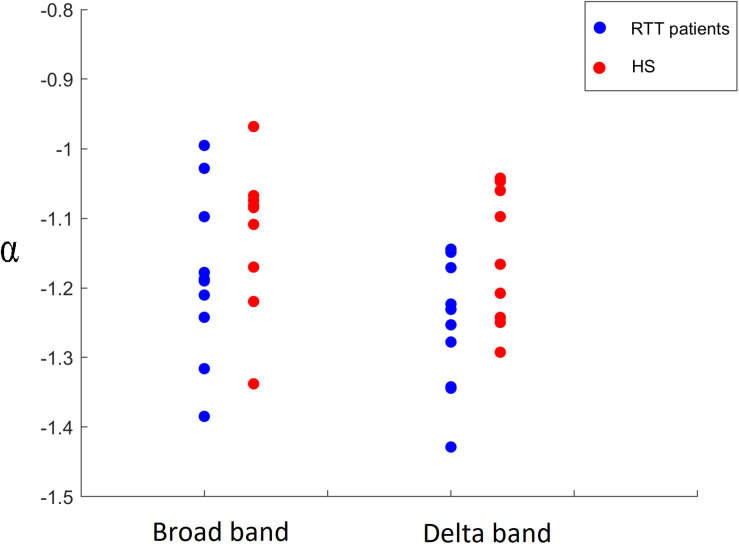
Comparison of α parameter in broadband for time bin Δ*t* = 2 and in delta band for time bin Δ*t* = 5 between RTT patients (blue dots) and HS (red dots).

Finally, a comparison of the α parameter between both groups was carried out, highlighting significant differences in the delta band (*p* = 0.0423) and in broadband (*p* = 0.0174), as shown in Figure 10. In particular, RTT patients invariably showed a lower distribution parameter α in both frequency bands, for all time bins. No significant differences were found in other frequency bands.

## Discussion

In this work, we set out to test the hypothesis that genetic mutations related to RTT would induce a reorganization of the whole-brain activity.

In particular, we characterized the distribution of neuronal avalanches in a group of 10 patients with Rett syndrome and 10 matched controls, using MEG, in an open-eye resting-state condition. The analysis was performed both in broadband and in the five canonical frequency bands. The study originated from recent evidence showing that the dynamics of resting-state brain activity, measured using MEG, produces scale-invariant neuronal avalanches, suggesting that the critical state is a physiological condition that is (presumably) optimal to the brain functioning. Consequently, the deviations from this optimally tuned configuration might convey the effects of the disease on large-scale coordinated activity (Meisel et al., 2012).

Our data are in line with recent findings showing that, in humans, the dynamics of resting-state brain activity is well described by a branching process where the sigma parameter is close to 1 (Meisel et al., 2013; Shriki et al., 2013).

Besides these confirmatory results, the main goal of this work is to study the frequency-specific differences in functional dynamics in RTT patients with respect to a control group. Our data showed that, around the critical state, the distribution size of neuronal avalanches, for both groups, obeys a power law. Interestingly, RTT patients show a lower value of the distribution parameter α both in delta and broadband, compared to controls. Furthermore, the exponents of the power law distributions vary greatly per each frequency band. This result is in line with Thompson et al., which suggests that resting-state connectivity is a frequency-dependent phenomenon (Thompson and Fransson, 2015).

The aforementioned results collectively suggest a global rearrangement in the brain functional dynamics of Rett patients. In fact, big avalanches capture the presence of widespread coordinated activations, with many different brain areas activating in a complex, meaningful sequence. The lack of large avalanches in patients might be a sign of the inability of the brain to coordinate a sufficient number of areas in sequence. One might speculate that the lack of large patterns of structured activity might be capturing the effects of the circuital changes induced by the genetic mutations present in RTT. This hypothesis might be compatible with the work by Armstrong et al., which claims that whole-brain structural changes are related to small, low-density neurons that have less dendritic branching and spine density (Armstrong et al., 1995). Furthermore, in the RTT syndrome, the dendrites of pyramidal neurons in the motor and frontal cortices are considerably shorter than in HS (Armstrong et al., 1995).

While our results are not conclusive about the brain being operating at criticality, they appear to be compatible with such hypothesis. This might be relevant given that it is known that critical dynamics facilitates optimization of processes such as information transfer and storage capacity. In particular, SOC involves a number advantages in information processing: (i) optimal information transmission because there is a perfect balance between the propagation of the signal over long distances and the resistance to saturation (Beggs and Plenz, 2003; Bertschinger and Natschläger, 2004; Kinouchi and Copelli, 2006); (ii) maximum mnemonic repertoire size, as the number of significant repetitive avalanche patterns is maximized (Haldeman and Beggs, 2005; Shew et al., 2011; Fagerholm et al., 2016); and (iii) maximized computational performance, as near the critical point, networks have better performances in different computational tasks, compared to networks with subcritical or supercritical dynamics (Bertschinger and Natschläger, 2004).

## Conclusion

Concluding, in our study we analyzed brain dynamics during eye-open resting state, measuring the size distribution of neuronal avalanches. We found a lower distribution parameter α in RTT patients in the delta frequency band and in the broadband, compared to controls. Such finding means that patients display a larger number of small avalanches and a lower number of big avalanches. These results can be explained in terms of reduced long-range coordination of neuronal activity across the brain in the pathological group. Such altered brain dynamics is likely to be suboptimal, and might underpin some of the symptomatology observed in Rett syndrome. Additionally, our results suggest that neuronal avalanches could be an innovative and advanced method to study the dynamics of brain activity. In fact, despite the rising interest in what constitutes the normal cortical dynamics in healthy humans, the nature of the alterations induced by pathological processes remains a major question in neuroscience. Interestingly, the observation that cortical networks optimize information processing when they are in a critical state might be exploited to identify subtle, preclinical brain dysfunction. Furthermore, the study of the critical dynamics of the human brain could be enhanced by comparing normal resting avalanches with evoked states or pharmacologically modified states and assessing the sensitivity (or performing a profile analysis) of the system with respect to the branching parameter values. Finally, such approaches are inherently multivariate, taking into account the activity of all brain areas at once, hence providing solid theoretical ground to the study of emerging, holistic properties of the brain.

## Data Availability Statement

The raw data supporting the conclusions of this article will be made available by the authors, without undue reservation.

## Ethics Statement

The studies involving human participants were reviewed and approved by “COMITATO ETICO UNIVERSITA’ FEDERICO II” n° protocol: 392/19. The patients/participants provided their written informed consent to participate in this study.

## Author Contributions

RR collected the sample, performed the MEG recordings, preprocessed the MEG data, contributed to MEG data analysis and statistical analysis, wrote the manuscript, and prepared the figures. PB collected the sample, contributed to clinical data analysis, and wrote the manuscript. AL performed the MEG recordings and preprocessed the MEG data. FB collaborated with the MEG data analysis and with the statistical analysis. MP performed the MEG recordings and preprocessed the MEG data. AP performed the MEG recordings and preprocessed the MEG data. CB collected the sample. CG, LM, and GS supervised the study. PS contributed to interpreting the results and critically revised the article. All authors read and approved the final version of the manuscript.

## Conflict of Interest

The authors declare that the research was conducted in the absence of any commercial or financial relationships that could be construed as a potential conflict of interest.

## Abbreviations

HS: healthy subjects
MECP2: methyl CpG-binding protein 2
MEG: magnetoencephalography
ROIs: regions of interests
RTT: Rett syndrome
SOC: self-organized criticality.
